# Turning Adversity into Strength and Transferring It to the Uninitiated: The Tricks Cancer Cells Play to Survive Hypoxic Stress and Fight Chemotherapy

**DOI:** 10.3390/cancers15010166

**Published:** 2022-12-28

**Authors:** Shashi Anand, Mohammad Aslam Khan, Ajay Pratap Singh

**Affiliations:** 1 Department of Pathology, College of Medicine, University of South Alabama, Mobile, AL 36617, USA; 2 Cancer Biology Program, Mitchell Cancer Institute, University of South Alabama, Mobile, AL 36604, USA; 3 Department of Biochemistry and Molecular Biology, College of Medicine, University of South Alabama, Mobile, AL 36688, USA

Despite significant progress during the past few decades, cancer remains the second most common cause of death in the US after heart disease. This year, we expect to observe about 1.9 million new cancer diagnosis and 609,360 deaths from cancer in the US alone [1]. Cancer, in fact, is the most debilitating word for many; however, the journey of a cancer cell from initial transformation to becoming an aggressive and life-threatening entity is not an easy one. During their evolution, cancer cells encounter many adversities, from immune attack to the scarcity of nutrients and oxygen and later facing the fight with therapeutic modalities. Despite these difficulties, cancer cells succeed in several individuals to become a clinically challenging disease by overcoming the challenges that they encounter. Thus, a precise understanding of the underlying mechanisms that allow relentless progression of cancer cells can help us develop effective approaches to manage cancer and improve the survival of patients.

Ovarian cancer (OC) is one of the deadliest cancers affecting women globally. Asymptomatic progression during the early stages and the lack of reliable biomarkers for screening result in its delayed diagnosis, limiting the options for therapy. Moreover, inherent or acquired resistance to chemotherapy is also common, leading to the therapeutic failure and frequent recurrence following an initial response [2,3]. Platinum-based drugs alone, or in combination with other anti-cancer drugs, are used as a primary line of therapy and often show promising response during the early treatment phase. However, disease relapse is common, and tumors come back in more aggressive forms for which no effective treatments are currently available [4,5]. Clearly, chemoresistance (innate or acquired) poses a major challenge in clinical management of ovarian malignancy, and a better understanding of the underlying mechanisms is greatly desired.

A recent study by Alharbi et al., published in Cancers [6], demonstrated a novel role of small-size extracellular vesicles (sEVs) in imparting OC chemoresistance by altering tumor cell metabolism. The authors show that sEVs-mediated chemoresistance occurs via transfer of hypoxia-induced glycolytic pathway proteins from hypoxic cells to the normoxic OC cells. Further, they display that sEVs isolated from a clinical cohort were also enriched in glycolysis-pathway proteins, especially in patients with recurrent disease. These exciting in vitro and clinical findings expose a novel mode of acquired chemoresistance in OC. Moreover, the data suggest that sEVs could serve as a useful tool for predicting the responsiveness of OC cells to chemotherapy beforehand and/or help in monitoring the therapeutic response during the treatment.

Extracellular vesicles (EVs) have emerged as a novel class of intercellular communicators that play significant roles in tumor progression, metastasis, and chemoresistance [7,8]. Evs released from the tumor cells can affect the local tumor microenvironment (TME), as well as help in pre-metastatic niche formation at distant organ sites [9]. Similarly, EVs from the host cells are also shown to alter tumor cell phenotypes and thus function as bi-directional communicators [10]. EVs harbor a variety of bioactive cargo, including coding and non-coding RNAs, DNA, and proteins from the donor cells. The transfer of this bioactive material to the recipient cells imparts changes in their behavior and display physiological and pathological consequences [11,12]. These recognitions have fueled the interest in EVs to investigate their multifaceted roles in tumor biology and to examine their clinical utility in diagnosis, prognosis, and therapy.

Hypoxia is a common feature of all solid malignancies that contributes to tumor aggressiveness and therapy resistance. It results from rapid proliferation of the tumor cells, desmoplastic reaction, and insufficient or abnormal tumor vasculature. Tumor cells adapt to the hypoxic stress by acquiring slow proliferating and invasive stem cell-like phenotypes, reprogramming their metabolism, limiting energy requirement, and release of angiogenic factors to renormalize the TME. Several studies, including ours, have exemplified that hypoxia affects the release, size distribution, and composition of EVs, which plays a significant role in the adaptation of tumor cells to the dynamic and challenging TME. Especially, tumor-derived sEVs are shown to exhibit alterations under hypoxic TME and to play a role in adaptive tumor cell survival and stromal remodeling [13,14,15]. Along the same line, the study by Alharbi et al. published in Cancers [6] demonstrates a novel role of sEVs in imparting OC chemoresistance via altering tumor cell metabolism.

In their rigorous study, Alharbi et al. used a panel of nine OC cell lines, of which the CAOV-3 cell line exhibited the least chemosensitivity to carboplatin under hypoxia. The MS/MS analysis identified thirty proteins that had significant differential expression in CAOV-3 cells upon their culturing in a hypoxic environment. Metabolism-related proteins, 3-hydroxyacyl-CoA dehydrogenase type-2, and succinate-CoA ligase were among the most enriched proteins in hypoxic OC cells. This is significant, as these two proteins have been associated with OC aggressiveness and platinum resistance [16,17]. Gene set enrichment analysis (GSEA) of proteomic data suggested the enrichment of proteins associated with hypoxia signaling, epithelial-to-mesenchymal transition (EMT), glycolysis, and MYC downstream signaling in hypoxic CAOV-3 cells. These pathways have been associated with hypoxia adaptive responses, as well as cancer chemoresistance [18,19]. The authors also found enhanced release of sEVs from these cells under hypoxia, which is in accordance with other prior observations in different cancer cell types [13,20].

Metabolic reprogramming is a key adaptive response of cancer cells when subjected to the low oxygen environment [19]. Authors report cell line-specific differential responses in the induction of glycolytic proteins. Interestingly, comparative analysis of differentially-expressed proteins in hypoxic OC cells and their derived hypoxic sEVs revealed specific packaging of certain proteins into sEVs, especially those associated with mitochondrial dysfunction and nuclear factor erythroid 2–related factor 2 (NRF2)-mediated oxidative stress response and synaptogenesis signaling pathways. It should be noted that alterations in mitochondrial function, including biogenesis, fission/fusion, and membrane dynamics have been associated with metabolic reprogramming during hypoxic stress as well as with chemoresistance in OC cells [21]. Similarly, NRF2 signaling, activated under hypoxia to alleviate oxidative stress, inhibits apoptosis, thereby promoting cell growth and chemoresistance [22]. In addition, dysregulation of proteins involved in metabolic reprogramming has been demonstrated to play an important role in the regulation of chemoresistance in other cancer cells as well [23].

Alharbi et. al. further examined whether sEVs released from hypoxic cells could confer chemoresistance to OC cells even when those cells were cultured under normoxia. Their data show that the treatment of CAOV-3 cells with sEVs released from hypoxic cells decreased their responsiveness to carboplatin-mediated apoptosis, even in normoxic environments. They further performed proteome analysis of the recipient cells to gain further mechanistic insight and identified 132 proteins that were significantly differentially expressed in cells incubated with hypoxic-sEVs than those treated with normoxic-sEVs. GSEA analysis revealed that these proteins were associated with oxygen–tension mechanisms, including hypoxia-inducible factors (HIFs), the glycolysis pathway, fatty acid synthesis, and protein secretion pathways. Thus, their data suggest that normoxic cells incubated with hypoxic sEVs attain a metabolic phenotype of hypoxic cells, which renders them resistant to carboplatin treatment. It would have been compelling to examine if hypoxic sEV treatment imparted stemness properties as well. Hypoxia-induced cancer stemness is suggested to be associated with the development of chemoresistance and poor prognosis [24]. In addition, in other studies, a role of sEVs released from hypoxic cancer cells has been reported in imparting stemness and invasive characteristics in non-hypoxic cancer cells [25,26].

Most importantly, in their study, Alharbi et. al. also analyzed sEVs isolated from the plasma of relapsed OC patients to gather supporting evidence from the clinical standpoint. Approximately 25% of the total proteins identified in plasma of cancer patients were also detected in sEVs from CAOV-3 cells cultured under limited oxygen conditions. More specifically, four proteins (pyruvate kinase M1/2, enolase-1, and aldolase fructose-bisphosphates) associated with glycolysis pathway were significantly higher in circulating sEVs of OC patients with disease recurrence compared to the non-cancer subjects. The role of these four proteins in cancer progression, metastasis, and chemoresistance is well documented in multiple cancers [27,28,29]. Therefore, studies in larger cohorts of patient samples should be pursued to analyze their significance in making prognostic assessment and predict the responsive of patients to platinum-based therapies. From the biological perspective, the impact of hypoxic sEVs on other cellular components of TME should also be examined, which could yield additional mechanistic insights into cancer evolution, adaptation, and gain of aggressive traits, as well as chemoresistance.

Taken together, it can be said that EVs are continuously emerging as important players in cancer pathobiology and as promising tools for disease diagnosis, prognosis, monitoring, and treatment strategies. The field is still evolving, but the potential is immense as we continue to advance in EV isolation and sorting, identification of donor cell-specific EVs markers, microfluidic-based analyses, etc. Furthermore, considering the key roles EVs play in tumor progression, metastasis, chemoresistance, pathways involved in their release, and uptake and specific loading of cargo could also be targeted for preventive and/or therapeutic interventions.
